# *In silico* Design of Linear DNA for Robust Cell-Free Gene Expression

**DOI:** 10.3389/fbioe.2021.670341

**Published:** 2021-05-18

**Authors:** Xinjie Chen, Yuan Lu

**Affiliations:** Key Laboratory of Industrial Biocatalysis, Ministry of Education, Department of Chemical Engineering, Tsinghua University, Beijing, China

**Keywords:** cell-free expression, cell-free protein synthesis, degradation inhibition, linear DNA expression template, rational design

## Abstract

Cell-free gene expression systems with linear DNA expression templates (LDETs) have been widely applied in artificial cells, biochips, and high-throughput screening. However, due to the degradation caused by native nucleases in cell extracts, the transcription with linear DNA templates is weak, thereby resulting in low protein expression level, which greatly limits the development of cell-free systems using linear DNA templates. In this study, the protective sequences for stabilizing linear DNA and the transcribed mRNAs were rationally designed according to nucleases’ action mechanism, whose effectiveness was evaluated through computer simulation and cell-free gene expression. The cell-free experiment results indicated that, with the combined protection of designed sequence and GamS protein, the protein expression of LDET-based cell-free systems could reach the same level as plasmid-based cell-free systems. This study would potentially promote the development of the LDET-based cell-free gene expression system for broader applications.

## Introduction

Cell-free synthetic biology (Garamella et al., 2016; Caschera, 2017; Damiati et al., 2018; Smolskaya et al., 2020) has rapidly developed as a powerful and flexible technology to overcome the inherent limitations of synthetic biology with living cells. By eliminating the constraint of sustaining life, cell-free systems provide unprecedented control over the molecular context for gene expression and metabolism (Silverman et al., 2020). Over the past 20 years, practical improvements (Dopp et al., 2019) in cell-free gene expression systems have seen its widespread adoption in basic research and industrial applications (Carlson et al., 2012; Silverman et al., 2020), such as genetic prototyping (Moore et al., 2017), artificial cells (Noireaux and Libchaber, 2004; Lai et al., 2020), high-throughput screening (Contreras-Llano and Tan, 2018), and biosensing (Verosloff et al., 2019). Compared with traditional cell-based approaches, one of the advantages of cell-free approaches is that linear DNA could be used as expression templates. The linear DNA could be mass-produced by PCR (polymerase chain reaction) (Schinn et al., 2016), which shortens the experimental period from 1 or 2 weeks to 1 or 2 days. Thus, there is a growing interest in studying cell-free gene expression systems with linear DNA expression templates (LDETs). Because of its natural configuration, linear DNA is more suitable for binding to materials than circular plasmids (Finkler and Ott, 2019). Currently, LDET-based cell-free systems have also been widely used in many aspects, such as biochip (Bar and Bar-Ziv, 2009; Heyman et al., 2012; Karzbrun et al., 2014), artificial cells (Xu et al., 2016), and high-throughput screening (Woodrow et al., 2006; Wu et al., 2007). The principle of these studies is to build cells from the bottom up, explore the origin of life, and promise industrial application.

However, compared with plasmid-based cell-free systems, LDET-based cell-free systems often suffer from low protein expression yield, which limits its further development. The main reason is that linear DNA can be easily degraded by native nucleases in cell extracts (Ahn et al., 2005). For example, in *Escherichia coli* cell extracts, RecBCD DNase complex (Klocke et al., 2018) has been shown to degrade linear DNA in the cell-free gene expression system. Now, researchers mainly overcome this problem in three ways. The first way is genomic alteration. Hong et al. (2015) tested five potential negative effectors to obtain more effective cell extract to improve cell-free protein synthesis. Unfortunately, genomic alteration always results in a slow growth rate and low cell viability, making preparing cell extracts difficult. The second way is finding DNase inhibitors. A kind of Gam protein from bacteriophage λ, GamS (Wilkinson et al., 2016b), has been found to be an effective inhibitor of RecBCD DNase complex (Sitaraman et al., 2004). However, the protein expression yield of LDET-based cell-free systems with GamS is only one-third of plasmid-based cell-free systems (Sun et al., 2014). There were also several studies focusing on DNA-binding proteins, such as Ku (Yim et al., 2020) and scCro (Zhu et al., 2020), to block the binding of native nucleases. There is still much room for improvement. Marshall et al. (2017) showed that short double-stranded DNA encoding chi sites could also be an efficient inhibitor. The third possible way is the alteration of template geometry. For example, the T7 terminator sequence, poly(G) sequence, and other non-coding sequences (Ahn et al., 2005) have been explored to improve the expression yield of LDET-based cell-free systems (Schinn et al., 2016). However, these studies only concentrate on adding additional inhibitors and ignore the potential of DNA sequence design on inhibiting the activity of native nucleases.

**FIGURE 1:**
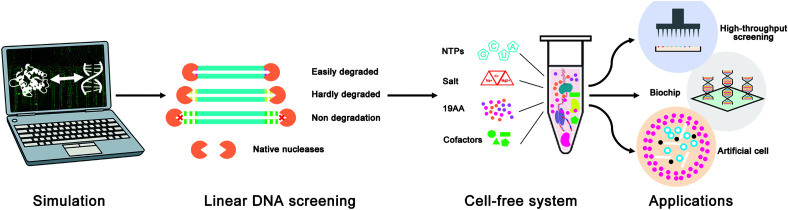
Schematic diagram of the linear DNA design strategy for the optimization of LDET-based cell-free systems. The computer simulation was used to evaluate the effectiveness of the linear DNA design strategy. Cell-free experiments were carried out to verify the results of the computer simulation. This design strategy could help to promote the development of cell-free systems for various applications.

In this study, a novel *in silico* design strategy was proposed to improve the stability of linear DNA in cell-free gene expression systems (Figure 1). The binding sites of native nucleases to linear DNA are always located at the end of linear DNA, so rationally designing protective sequences at the end of linear DNA can be an effective way to protect linear DNA from native nucleases. Furthermore, both computer simulation and cell-free gene expression experiments were used to evaluate the effectiveness of the linear DNA design strategy. It is hoped that this design strategy would be a useful and convenient way to improve the expression yield of LDET-based cell-free systems and promote the development of cell-free synthetic biology.

## Results and Discussion

### Design Strategy for GC Content in Protective Sequence

**FIGURE 2:**
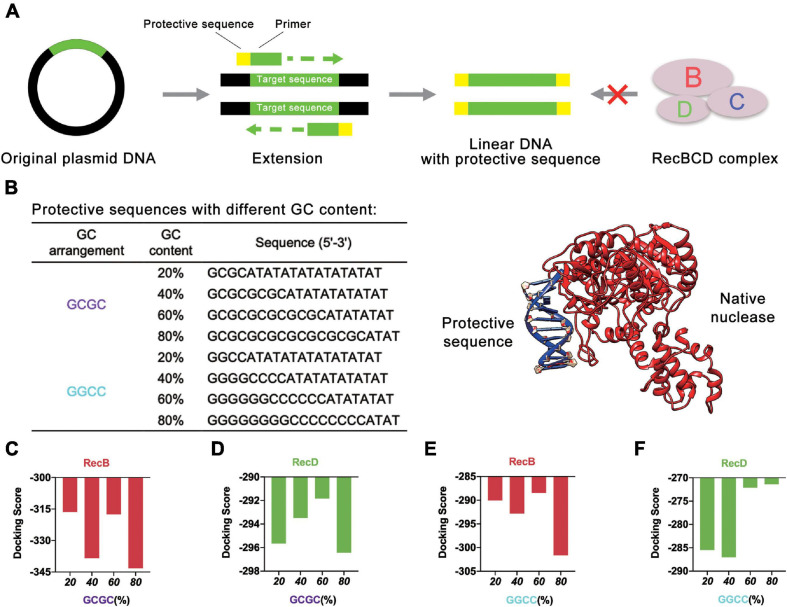
Protective sequences with different GC content. **(A)** The protective sequences were added to the ends of linear DNA by PCR. The protective sequences could protect linear DNA from the degradation of native nucleases. **(B)** Protective sequences with different GC arrangement and GC content. GC arrangement included GCGC and GGCC. GC content included 20, 40, 60, and 80%. The length of protective sequences was 20 bp. The diagram on the right side was native nuclease binding with the protective sequence. **(C)** Simulation results of RecB subunit with different protective sequences. These protective sequences were GCGC arrangement mode and had different GC contents. **(D)** Simulation results of RecD subunit with different protective sequences. These protective sequences were GCGC arrangement mode and had different GC contents. **(E)** Simulation results of RecB subunit with different protective sequences. These protective sequences were GGCC arrangement mode and had different GC contents. **(F)** Simulation results of RecD subunit with different protective sequences. These protective sequences were GGCC arrangement mode and had different GC contents.

Initially, different protective sequences with different GC contents were designed to explore their effects on the stability of linear DNA. At present, the most common way to obtain linear DNA was by PCR. In this study, the protective sequence was amplified to the end of the linear DNA by designing primers with a protective sequence (Figure 2A). The first principle of the protective sequence design was that the GC content affected the stability of linear DNA (Woodrow et al., 2006), so protective sequences were designed with different GC contents to explore the best GC content. Moreover, considering that an excessively long primer would lead to higher reaction temperatures and make PCR more difficult, the length of the protective sequence was limited to 20 bp. If the length of the protective sequence was more than 20 bp, there might be a mismatch, and the PCR product would be multiple. The PCR results proved that when the length of the protective sequence was 20 bp, linear DNA was constructed well (Supplementary Figure 1). At the same time, to reduce the effect of GC arrangement, two sets of protective sequences were designed with different GC arrangement modes (Figure 2B). One was “GCGC” arrangement mode, and the other was “GGCC” arrangement mode.

The computer simulation was used to evaluate the design strategy, and *DNA Sequence to Structure* webserver (Arnott et al., 1976) was finally selected for predicting and modeling the 3D structure of the target linear DNA sequence. Another webserver, *HDOCK SERVER* (Huang and Zou, 2008, 2014; Yan et al., 2017a, b, 2020), was used to evaluate the binding efficiency of RecBCD DNase complex with the designed linear DNA. The RecBCD DNase complex is composed of three different subunits called RecB, RecC, and RecD. Among these subunits, RecB subunit was 3′-5′ helicase, RecC subunit recognized *Chi* sequence, and RecD subunit was 5′-3′ helicase (Wilkinson et al., 2016a). According to this principle, RecB subunit and RecD subunit (Supplementary Table 1 and Supplementary Figures 2A,B) were selected for simulation (Figures 2C–F and Supplementary Figure 3). Combined with the results of the four groups of experiments, it could be concluded that when the GC content of the protective sequence was close to 60%, the docking scores of RecB subunit and RecD subunit with the designed linear DNAs were the highest in each group. A higher docking score represented a worse docking efficiency, which meant it was difficult for RecBCD DNase complex docking with linear DNA. In addition, it was also found that no matter what the GC arrangement method was, in the simulation results of linear DNA with RecD subunit, the gap between different docking scores was far smaller than the results of linear DNA with RecB subunit. This finding showed that RecB subunit was more sensitive to the change of the protective sequence, which indicated that RecB subunit was more suitable to represent the docking efficiency of linear DNA with RecBCD DNase complex. The results also showed that the different docking scores of “GCGC” groups were all lower than those of “GGCC” groups, which meant that the interaction between RecBCD DNase complex and “GGCC” groups was much weaker.

**FIGURE 3:**
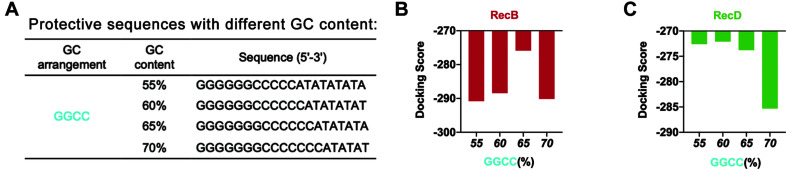
Simulation results of protective sequences with different GC contents. **(A)** Protective sequences with different GC contents. GC contents included 55, 60, 65, and 70%. The length of protective sequences was 20 bp. **(B)** Simulation results of RecB subunit with different protective sequences. These protective sequences were GGCC arrangement mode and had different GC contents. **(C)** Simulation results of RecD subunit with different protective sequences. These protective sequences were GGCC arrangement mode and had different GC contents.

Based on this point, to further optimize the GC content of the protective sequences, the GC content was further refined (Figure 3A). The docking simulation results showed that when GC content was 65%, the docking score of linear DNA and RecB subunit was the highest (Figure 3B and Supplementary Figure 4A). When the GC content was between 55 and 65%, the docking score of linear DNA and RecD subunit was significantly higher (Figure 3C and Supplementary Figure 4B). In this case, the docking score of linear DNA and RecB was −275.89, while the docking score of normal linear DNA and RecB subunit was −319.04. This result indicated that the protective sequence did improve the stability of linear DNA. The reason was that the increase of GC content could increase the number of hydrogen bonds between DNA double strands, to enhance the stability of linear DNA and increase the difficulty of native nucleases binding. It should be noted that since the length of the protective sequence was only 20 bp, the variation of only one base pair could result in a 5% GC content change. It was difficult to further optimize the GC content of the protective sequences. In this part, by comprehensively analyzing the docking simulation results of linear DNA and two subunits, it was concluded that the best GC content was between 60 and 65%.

### Design Strategy for GC Distribution in Protective Sequence

To further optimize the protective sequences, the distribution of GC sequences was worthy of exploration. In the previous docking simulation, to avoid the interference of GC distribution on the simulation results, GC sequences were uniformly distributed at the front end of the designed linear DNA. Since the best GC content was between 60 and 65%, 60 and 65% GC content were selected for further docking simulation. In addition, considering that GC arrangement might also affect the docking between linear DNA and nucleases, both “GCGC” and “GGCC” arrangement modes were still adopted in the design process. The previous docking simulation had confirmed that RecB subunit was more suitable to characterize the docking efficiency of linear DNA and nucleases. Therefore, only the docking efficiency of linear DNA and RecB subunit was simulated to reduce the workload.

**FIGURE 4:**
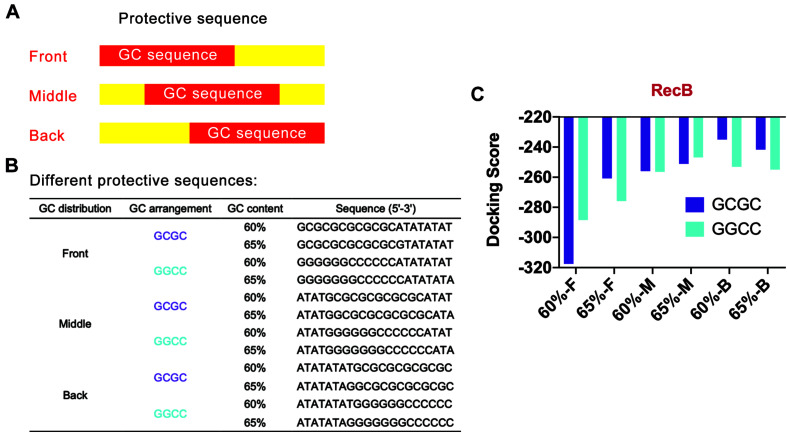
Simulation results of protective sequences with different GC distributions. **(A)** Schematic diagram of protective sequences with different GC distributions. The GC distribution modes included front, middle, and back. **(B)** Protective sequences with different GC distributions, arrangements, and contents. The length of protective sequences was 20 bp. **(C)** Simulation results of RecB subunit with different protective sequences. The 60% and 65% meant different GC contents. The letter F, M, and E meant the GC distribution mode was front, middle, and back. These protective sequences had two different GC arrangement modes, including GCGC and GGCC.

Based on the above points, several protective sequences with different GC distribution modes were designed (Figures 4A,B). *DNA Sequence to Structure* webserver was still used to predict and model the 3D structure of the target linear DNA sequence. *HDOCK SERVER* was used to evaluate the binding efficiency of RecB subunit with the designed linear DNA (Figure 4C and Supplementary Figure 5). From the perspective of GC distribution mode, it was found that, compared with the four groups where GC sequences were distributed in the front of the protective sequence, the docking scores of other groups with GC sequences distributed in the middle or the back of the protective sequence were higher, which meant more difficulty for RecB subunit docking with linear DNA. From the perspective of GC content, when GC sequences were distributed in the middle or the back of the protective sequence, the docking simulation results of each group with GC content of 60% were not significantly different from those with GC content of 65%. However, when GC sequences were distributed in the front of the protective sequence, the docking scores of groups with 65% GC content were higher than those with 60% GC content. Furthermore, from the perspective of GC arrangement mode, the results showed that there was no significant relationship between the docking score and GC arrangement.

According to the above docking simulation results, the current optimal GC distribution mode was that the GC sequence was located at the middle or the back of the protective sequence. It was worth noting that when the GC distribution mode was back and the GC arrangement was GCGC, the docking score was −235.06, while the normal docking score was −319.04. The results indicated that the protective sequence could greatly reduce the docking efficiency of linear DNA with nucleases and improve the stability of linear DNA.

### Design Strategy for Stem-Loop Structure at 3′ End of mRNA

**FIGURE 5:**
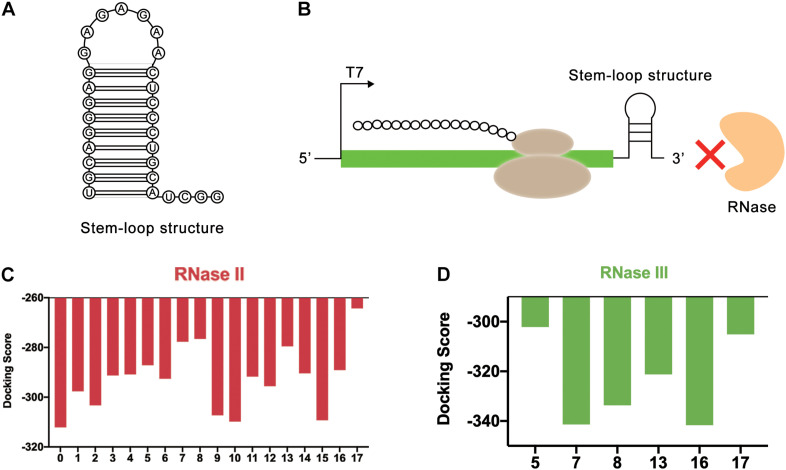
REP sequences could form a stem-loop structure to block the binding of RNase. **(A)** The secondary structure of stem-loop structure formed by REP sequence. **(B)** The stem-loop structure could block the end of mRNA. In this way, the REP sequence could protect mRNA from the degradation of RNase. **(C)** Simulation results of RNase II and REP sequences. The numbers on the abscissa represented different REP sequences, and the number 0 represented the control group without REP sequence. **(D)** Simulation results of RNase III and REP sequences. The numbers on the abscissa represented different REP sequences.

In the LDET-based cell-free system, the expression yield of the target protein was restricted by two factors. One was that linear DNA was easy to degrade with native nucleases. The other was that mRNA could be easily degraded by RNase, resulting in a short duration of translation and low expression yield. In the previous sequence design process, the protective sequence had been designed for the first case. Therefore, further sequence design would be developed for the second case. In cell-free gene expression systems, RNase mainly included RNase II, RNase III (Court et al., 2013), PNPase, and RNase E. All of them were 3-terminal exonuclease (Łabno et al., 2016) except RNase E (Grunberg-Manago, 1999). Poly(G) sequence had been proven to inhibit the activity of PNPase well (Ahn et al., 2005). Therefore, RNase II and RNase III (Supplementary Table 1 and Supplementary Figures 2C,D) were selected for simulation in the end. In 2018, Deng et al. used synthetic repetitive extragenic palindromic (REP) sequences as an effective mRNA stabilizer in two typical prokaryotic microbes (Deng et al., 2019). REP sequence was a kind of regulatory sequence located in the untranslated operon region in most bacteria, which could form a stable stem-loop structure based on its palindromic properties (Deng et al., 2019). Therefore, it could be a great attempt to introduce the REP sequence (Liang et al., 2015) into the linear gene template for the cell-free gene expression system. Based on the action mechanism of degrading enzymes, REP sequences were added to 3′ end of mRNA to improve mRNA stability. The design of REP sequences could form a stem-loop structure to block the binding of RNase (Figures 5A,B).

In this study, the effectiveness of REP sequences was evaluated by computer simulation, which improved the screening efficiency. Considering that different REP sequences would form different stem-loop structures with different lengths, the effect of the length of the stem-loop structure was ignored at first. Only the length of the mRNA sequence used for simulation was kept the same. To achieve this, the REP sequences were attached to the end of the mRNA. In total, 17 different REP sequences from natural *E. coli* gene sequences with single stem structures were selected for docking simulation (Supplementary Table 2). These REP sequences were all selected from *RNAstem* database^1^. *Vfold2D* webserver (Cao and Chen, 2005, 2009; Xu et al., 2014; Xu and Chen, 2016) was used to predict the secondary structure of the designed mRNA sequences, and *3dRNA v2.0* webserver (Wang et al., 2015, 2017, 2019) was used to construct the 3D structure of mRNA. At last, *HDOCK SERVER* was still used to simulate and evaluate the docking status between mRNA and RNase II. The results indicated that after adding REP sequences, the docking efficiency of mRNA and RNase II had been decreased, which meant the stem-loop structure improved the stability of mRNA (Figure 5C and Supplementary Figure 6). Among these sequences, six different sequences (Number 5, 7, 8, 13, 16, and 17), whose docking scores were all higher than −290, were selected for the following docking simulation with RNase III (Figure 5D and Supplementary Figure 7). Finally, it was found that the sequence number 17 had the highest docking score with the two RNases, which was later used to optimize the length of the stem-loop structure.

**FIGURE 6:**
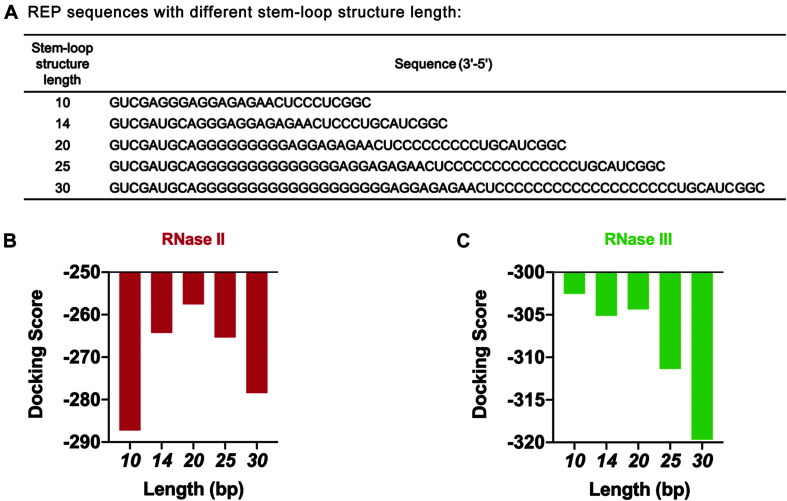
Simulation results of REP sequences with different stem-loop structure lengths. **(A)** REP sequences with different stem-loop structure lengths. The model REP sequence was the sequence number 17. **(B)** Simulation results of RNase II with different REP sequences. These REP sequences had different lengths, which ranged from 10 to 30 bp. **(C)** Simulation results of RNase III with different REP sequences. These REP sequences had different lengths, which ranged from 10 to 30 bp.

The length of the stem-loop structure was further adjusted by increasing the number of G and C bases (Figure 6A). The design principle was that the number of hydrogen bonds between GC base pairs was three, which was larger than the number of hydrogen bonds between AU base pairs. The stem-loop structure formed was more stable this way. *HDOCK SERVER* was used to simulate and evaluate the docking status between mRNA and two different RNases (Figures 6B,C and Supplementary Figure 8). As seen from the simulation results, when the length of the stem-loop structure was 20 bp, the docking scores of mRNA sequences and the two RNases were the highest. In the case where the stem-loop structure was too long or too short, the docking score of mRNA and RNase decreased, and the stability of mRNA decreased. However, the existence of the stem-loop structure might affect the process of translation and decrease the protein expression level of the cell-free gene expression system. Therefore, the designed REP sequence needed to be verified in the following experiments.

### Cell-Free Experimental Verification of Protective Sequence Design

**FIGURE 7:**
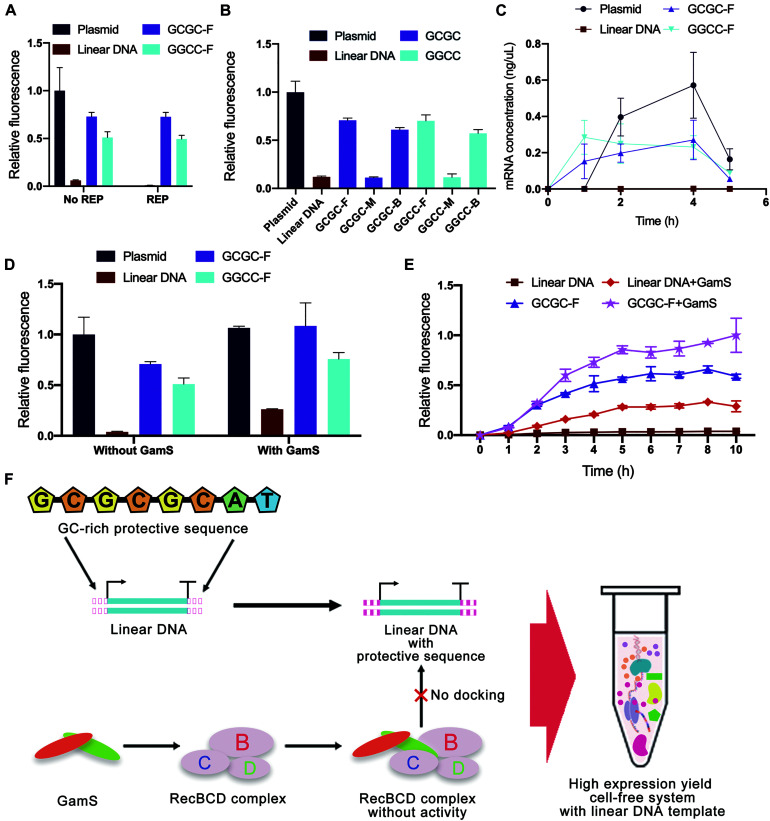
Cell-free experimental verification of protective sequence design. **(A)** Comparison of typical templates and linear templates with REP sequence. The REP sequence used was the REP sequence number 17. Only the plasmid group did not add the REP sequence. Linear DNA meant the linear expression templates did not add protective sequences. The GCGC meant protective sequences with GCGC arrangement mode were added to the linear expression templates. The GGCC meant protective sequences with GGCC arrangement mode were added to the linear expression templates. The GC distribution mode of these protective sequences was front. The fluorescence of the cell-free system with plasmid was set to 1. Error bars represented standard deviations from three replicates. **(B)** Experimental results of different expression templates in cell-free systems. The letters F, M, and E meant GC distribution mode was front, middle, and back. The fluorescence of the cell-free system with plasmid was set to 1. Error bars represented standard deviations from three replicates. **(C)** The kinetics of cell-free reactions with different expression templates. Error bars represented standard deviations from three replicates. **(D)** Comparison of sequence-based protection and GamS-based inhibition. The fluorescence of the cell-free system with normal plasmid was set to 1. Error bars represented standard deviations from three replicates. **(E)** Time curves of relative fluorescence for different protection strategies. The GC distribution mode of these protective sequences was front. The fluorescence of the cell-free system with the co-protection of protective sequence and GamS was set to 1. Error bars represented standard deviations from three replicates. **(F)** The co-protection of protective sequence and GamS inhibited the degradation from RecBCD complex. With the co-protection of the designed sequence and GamS, the protein expression yield of LDET-based cell-free systems could reach the same level as the plasmid-based cell-free systems.

Considering that the computer simulation results might be different from the actual binding of linear DNA to native nucleases in the cell-free gene expression system, a cell-free experiment was carried out to verify the effectiveness of *in silico* design of linear DNA. In this study, the green fluorescent protein sfGFP (Supplementary Figure 9) was used as a model protein, and the fluorescence of sfGFP was used to represent the protein expression yield. Different from the simulation results of REP sequences, the experimental results showed that there was no significant improvement in protein expression yield after adding the REP sequence (Figure 7A). The unexpected results might be attributed to two possible reasons. One reason was that the simulation result did not match the actual situation, and the other reason was that the existence of a stem-loop structure might negatively affect the whole translational process.

In contrast, the results also indicated that the addition of GC-rich sequences was highly efficient. Therefore, cell-free experiments were carried out to verify the results of GC-rich sequence design further. There were some differences between the experimental results and simulation results of GC-rich sequence design (Figure 7B). Previous simulation results showed that the optimal GC distribution mode was that the GC sequence was located at the back of the protective sequence. However, the results showed that, compared with the groups where GC sequences were distributed in the middle of the protective sequence, the sfGFP expression of other groups with GC sequences distributed in the front and back of the protective sequence was much higher. It seemed that distributing GC sequences in the front of the protective sequence was the best choice. The differences between simulation results and experimental results were mainly due to the errors generated during the simulation. Parameters used in simulation could not completely reflect the actual situation. Nevertheless, *in silico* design still provided very effective guidance for the inhibition of linear DNA degradation. Combining simulation and experimental results, the protein expression yield of linear DNA with protective sequence had reached 75% of that of plasmid and was six times of that of normal linear DNA. It indicated that the protective sequence could indeed greatly improve the expression yield of the LDET-based cell-free system. This phenomenon was because a protective sequence could effectively decrease the docking efficiency of linear DNA and RecBCD DNase complex, thereby ensuring the integrity of the gene expression template in the cell-free gene expression system. To prove this point, additional nucleases were added to the cell-free gene expression system (Supplementary Figure 10). After adding additional nucleases, the protein expression yield of the cell-free system with normal linear DNA decreased a lot, while there was still considerable expression in the groups with protective sequences. This result proved that protective sequences decreased the degradation of linear gene expression templates in the cell-free gene expression system.

To investigate whether the protective sequence affected the kinetics of cell-free reactions, the change of mRNA level in the cell-free gene expression system was detected with the increase of reaction time (Figure 7C). Compared to the typical linear DNA, the groups with a protective sequence had an obviously higher mRNA level. This result indicated that after the addition of the protective sequence, the transcription level of the cell-free gene expression system was greatly improved. However, there was still a gap between linear DNA with a protective sequence and the plasmid.

GamS is a common RecBCD DNase complex inhibitor. The mechanism involved saw GamS bind to RecBCD DNase complex to prevent the binding of complex and linear DNA (Wilkinson et al., 2016b). Therefore, the comparative cell-free experiment of sequence-based protection and GamS-based inhibition was carried out to explore the protective mechanism (Figure 7D). The results showed that the protein expression yield of plasmid-based cell-free systems was not changed with GamS. However, the protein expression yield of the group with typical linear DNA was slightly improved with GamS, which was lower than that with protective sequences. This comparison proved the effectiveness of our protective sequence design. There was also a further interesting finding. With the co-protection of the designed sequence and GamS, the protein expression yield of LDET-based cell-free systems could reach the same level as the plasmid-based cell-free systems. A further experiment was carried out to prove the co-protection of the designed sequence and GamS (Figure 7E). The overall expression level showed that, after 3 h of reaction, the expression level of the cell-free gene expression system with typical linear DNA template reached the peak, indicating that the linear DNA had been completely degraded, and the cell-free reaction stopped. However, with the co-protection of the designed sequence and GamS, the duration of the LDET-based cell-free system was longer, and the expression level was much higher. These results showed that the co-protection of the designed sequence and GamS could effectively inhibit the degradation of linear DNA in cell-free gene expression systems (Figure 7F).

## Conclusion

In this study, an *in silico* linear DNA design strategy was proposed to improve the stability of linear DNA in cell-free gene expression systems. The computer simulation was used to judge the effectiveness of different design strategies and provide guidance for the experiments. These strategies included the rational design of GC content, GC arrangement, GC distribution, and stem-loop structure. The simulation results not only helped determine the best GC content and stem-loop structure, but also provided a general direction for GC arrangement and GC distribution design. Later, by amplifying the protective sequence to the end of the linear DNA through primer design, the cell-free experiment results showed that the best GC content was between 60 and 65%, and distributing GC sequences in the front of the protective sequence was the best choice. This sequence design strategy improved the protein expression yield of the LDET-based cell-free system to 75% of that of the plasmid. Furthermore, with the co-protection of the designed sequence and GamS, the protein expression yield of the LDET-based cell-free system had reached the same level as that of the plasmid. These results not only highlighted the importance of DNA sequence design strategy on linear DNA stability, but also provided a novel method combining two different strategies to co-protect linear DNA from the degradation of native nucleases. This work could also promote the development of LDET-based cell-free systems and expand its application, such as in artificial cells, biochips, and high-throughput screening.

## Materials and Methods

### Simulation Tools

#### Three-Dimensional Structure of DNA

This study used *DNA Sequence to structure* webserver (Arnott et al., 1976)^2^ to predict the 3D structure of DNA sequences and build the 3D model.

#### Secondary Structure of RNA

This study used *Vfold2D (version2.0): Predicting RNA 2D structures* webserver (Cao and Chen, 2005, 2009; Xu et al., 2014; Xu and Chen, 2016)^3^ to predict the secondary structure of RNA.

#### Three-Dimensional Structure of RNA

This study used *3dRNA v2.0: Automatic building of ncRNA 3D structures* webserver (Wang et al., 2015, 2017, 2019)^4^ to build the 3D model of RNA.

#### Protein-DNA/RNA Docking

This study used *HDOCK SERVER* (Huang and Zou, 2008, 2014; Yan et al., 2017a, b, 2020)^5^ to simulate the docking between DNA and protein or RNA and protein.

#### Three-Dimensional Structure Visualization

This study used iCn3D (I-see-in-3D) (Wang et al., 2020), a web-based three-dimensional structure browser^6^, for visualization of the simulation results.

### Plasmid DNA and Linear DNA Preparation

Plasmids used in this study were performed following standard molecular biology techniques. The green fluorescent protein pET-23a-sfGFP (Supplementary Figure 9) was used as a model protein. The sequences of plasmids were verified by TianYi Biotechnology (Beijing, China). Linear DNA used in this study was produced by PCR. PCR reagents were from Beyotime (Shanghai, China). Several primers were designed for the synthesis of linear DNA (Supplementary Table 3). First, all reaction components (50μ*L* reaction) were assembled on ice. These components included 38μ*L* ddH_2_O, 5μ*L* 10× pfu buffer, 1μ*L* 10 mM dNTPs, 1.75μ*L* template DNA, 2μ*L* 10μ*M* forward primer, 2μ*L* 10μ*M* reverse primer, and 0.25μ*L* pfu polymerase. PCR tubes were transferred from ice to a PCR machine with the block preheated to 94°C, and thermocycling began. The program was run at 94°C for 3 min, followed by 30 cycles of 94°C for 30 s, 57°C for 30 s, and 72°C for 140 s. The final extension was run at 72°C for 5 min and 4°C for the remaining time. PCR product was mixed with 0.1 volume of sodium acetate (3 mol/L, pH = 5.2). Two volumes of ethanol were added to the sample and were frozen at −20°C for at least 1 h or overnight for best results. The sample was centrifuged at full speed for 20 min to collect all materials. The sample was washed with 70% ethanol. After that, the sample was centrifuged for 10–15 min to pellet the DNA. The ddH_2_O was added to dissolve DNA. DNA was stored at −20°C for standby use.

### Overlap PCR

Linear DNA with REP sequence was produced by overlap PCR. First, all reaction components were assembled (50μ*L* reaction) on ice. These components included 10μ*L* 5× Q5 buffer, 10μ*L* high GE enhancer, 1μ*L* 10 mM dNTPs, 50 ng forward template, 50 ng reverse template, 0.5μ*L* Q5 polymerase, and ddH_2_O. PCR tubes were transferred from ice to a PCR machine with the block preheated to 98°C, and thermocycling was begun. The program was run at 98°C for 30 s, followed by 15 cycles of 98°C for 10 s, 57°C for 30 s, and 72°C for 33 s. Forward template and reverse template were added for another 20 cycles. The final extension was run at 72°C for 2 min and 4°C for the remaining time. The PCR product was mixed with 0.1 volume of sodium acetate (3 mol/L, pH = 5.2). 2 volumes of ethanol were added to the sample and were frozen at −20°C for at least 1 h or overnight for best results. The sample was centrifuged at full speed for 20 min to collect all material. The sample was washed with 70% ethanol. After that, the sample was centrifuged for 10–15 min to pellet the DNA. The ddH_2_O was added to dissolve DNA. DNA was stored at −20°C for standby use.

### GamS Protein Purification

The composition of buffers used was as follows: buffer A (per liter), 29.22 g NaCl, an 2.422 g Tris, with pH set to 7.4 with hydrochloric acid; buffer B (per liter), 29.22 g NaCl, 2.422 g Tris, and 34 g imidazole, with pH set to 7.6 with hydrochloric acid. A frozen stock of GamS in a BL21(DE3) *E. coli* strain (Supplementary Figure 11) was grown overnight in LB-carbenicillin media. 20 mL was used to inoculate 1 L LB-carbenicillin to an OD 600 nm of 0.6–0.8 at 37°C, 220 rpm. 0.1% IPTG (Isopropyl-beta-D-thiogalactopyranoside) was added, and cells were grown for four additional hours at 37°C, 220 rpm. Cells were resuspended in buffer A, mechanically lysed, and purified with gravity flow columns (His GraviTrap, GE Healthcare). 25% sucrose was added, and protein was stored at −80°C for further use.

### Cell-Free Reactions

The cell-free reaction mixture included 1.5 mM spermidine, 1 mM putrescine, 0.33 mM NAD, 1.2 mM ATP, 0.86 mM CTP and GTP, 0.86 mM UTP, 0.27 mM Coenzyme A (CoA), 170 μg/mL tRNA, 34 μg/mL folinic acid, 33 mM phosphoenolpyruvate (PEP), 2 mM of each of the 19 amino acids, 175 mM potassium glutamate, 10 mM ammonium glutamate, 2.7 mM potassium oxalate, 1 mM Mg^2+^, 4 mM GSSG, 1 mM GSH, 2.5% PEG8000, and 30% (volume) of *E. coli* extract (Rosetta DE3). Cell-free reactions (20 μL) were incubated at 30°C for 16 h. The fluorescence was determined by the enzyme-labeled instrument. The excitation wavelength was 485 nm, and the absorptive wavelength was 535 nm. Because the fluorescence of sfGFP increased linearly with the concentration of protein, this was consistent with the result of Western blot (Supplementary Figure 12). In panel A of Figure 7, the relative fluorescence 1 meant 755240 A.U. fluorescent intensity, which meant the protein concentration was 0.76 mg/mL. In panel B of Figure 7 the relative fluorescence 1 meant 525740 A.U. fluorescent intensity, which meant the protein concentration was 0.53 mg/mL. In panel D of Figure 7, the relative fluorescence 1 meant 602250 A.U. fluorescent intensity, which meant the protein concentration was 0.61 mg/mL. In panel E of Figure 7, the relative fluorescence 1 meant 603540 A.U. fluorescent intensity, which meant the protein concentration was 0.61 mg/mL.

### RNA Extraction and Target mRNA Quantification

The total mRNA was extracted from the samples by RNAsimple Total RNA Kit (TIANGEN, DP419). The kit was stored at −80°C, or the reverse transcription was performed immediately by FastKing RT Kit (With gDNase) (TIANGEN, KR116). The cDNAs were quantified by SuperReal PreMix Plus (SYBR Green) (TIANGEN, FP205-02). The PCR product of sfGFP was amplified by the same primers as qPCR for the standard curve (Supplementary Figure 13) to relate the sfGFP cDNA concentrations with CT values read by ABI 7300 Real-Time PCR system.

## Data Availability Statement

The original contributions presented in the study are included in the article/Supplementary Material, further inquiries can be directed to the corresponding author.

## Author Contributions

YL conceived the project, supervised the research, assisted in analyzing the data, prepared the figures, and wrote the manuscript. XC designed and performed the experiments, analyzed the results, prepared the figures, and wrote the manuscript. Both authors contributed to the article and approved the submitted version.

## Conflict of Interest

The authors declare that the research was conducted in the absence of any commercial or financial relationships that could be construed as a potential conflict of interest.

## Footnotes

1 http://bioinformatics.bc.edu/meyerlab/RNAstem

2 http://scfbio-iitd.res.in/software/drugdesign/bdna.jsp

3 http://rna.physics.missouri.edu/vfold2D/index.html

4 http://biophy.hust.edu.cn/3dRNA

5 http://hdock.phys.hust.edu.cn/

6 https://www.ncbi.nlm.nih.gov/Structure/icn3d/full.html
