# Supplementary material for: In silico Design of Linear DNA for Robust Cell-Free Gene Expression
Source: Front Bioeng Biotechnol. 2021 May 18;9:670341. doi: 10.3389/fbioe.2021.670341 (PMC8169995; doi:10.3389/fbioe.2021.670341)
Supplement: Supplementary file 1 [file Data_Sheet_1.docx]

**Supplementary Material**

***In silico* design of linear DNA for robust cell-free gene expression**

Xinjie Chen^1^, Yuan Lu^1*^

^1^Key Laboratory of Industrial Biocatalysis, Ministry of Education, Department of Chemical Engineering, Tsinghua University, Beijing 100084, China.

^*^ Corresponding author. E-mail address: yuanlu@tsinghua.edu.cn (Yuan Lu)

# Table S1. The amino acid sequence of degrading enzymes used in this study

| Enzyme | Amino acid sequence |
| --- | --- |
| RecB | GMSDVAETLDPLRLPLQGERLIEASAGTGKTFTIAALYLRLLLGLGGSAAFPRPLTVEELLVVTFTEAATAELRGRIRSNIHELRIACLRETTDNPLYERLLEEIDDKAQAAQWLLLAERQMDEAAVFTIHGFCQRMLNLNAFESGMLFEQQLIEDESLLRYQACADFWRRHCYPLPREIAQVVFETWKGPQALLRDINRYLQGEAPVIKAPPPDDETLASRHAQIVARIDTVKQQWRDAVGELDALIESSGIDRRKFNRSNQAKWIDKISAWAEEETNSYQLPESLEKFSQRFLEDRTKAGGETPRHPLFEAIDQLLAEPLSIRDLVITRALAEIRETVAREKRRRGELGFDDMLSRLDSALRSESGEVLAAAIRTRFPVAMIDEFQDTDPQQYRIFRRIWHHQPETALLLIGDPKQAIYAFRGADIFTYMKARSEVHAHYTLDTNWRSAPGMVNSVNKLFSQTDDAFMFREIPFIPVKSAGKNQALRFVFKGETQPAMKMWLMEGESCGVGDYQSTMAQVCAAQIRDWLQAGQRGEALLMNGDDARPVRASDISVLVRSRQEAAQVRDALTLLEIPSVYLSNRDSVFETLEAQEMLWLLQAVMTPERENTLRSALATSMMGLNALDIETLNNDEHAWDVVVEEFDGYRQIWRKRGVMPMLRALMSARNIAENLLATAGGERRLTDILHISELLQEAGTQLESEHALVRWLSQHILEPDSNASSQQMRLESDKHLVQIVTIHKSKGLEYPLVWLPFITNFRVQEQAFYHDRHSFEAVLDLNAAPESVDLAEAERLAEDLRLLYVALTRSVWHCSLGVAPLVRRRGDKKGDTDVHQSALGRLLQKGEPQDAAGLRTCIEALCDDDIAWQTAQTGDNQPWQVNDVSTAELNAKTLQRLPGDNWRVTSYSGLQQRGHGIAQDLMPRLDVDAAGVASVVEEPTLTPHQFPRGASPGTFLHSLFEDLDFTQPVDPNWVREKLELGGFESQWEPVLTEWITAVLQAPLNETGVSLSQLSARNKQVEMEFYLPISEPLIASQLDTLIRQFDPLSAGCPPLEFMQVRGMLKGFIDLVFRHEGRYYLLAYKSNWLGEDSSAYTQQAMAAAMQAHRYDLQYQLYTLALHRYLRHRIADYDYEHHFGGVIYLFLRGVDKEHPQQGIYTTRPNAGLIALMDEMFAGMTLEEA |
| RecD | MKLQKQLLEAVEHKQLRPLDVQFALTVAGDEHPAVTLAAALLSHDAGEGHVCLPLSRLENNEASHPLLATCVSEIGELQNWEECLLASQAVSRGDEPTPMILCGDRLYLNRMWCNERTVARFFNEVNHAIEVDEALLAQTLDKLFPVSDEINWQKVAAAVALTRRISVISGGPGTGKTTTVAKLLAALIQMADGERCRIRLAAPTGKAAARLTESLGKALRQLPLTDEQKKRIPEDASTLHRLLGAQPGSQRLRHHAGNPLHLDVLVVDEASMIDLPMMSRLIDALPDHARVIFLGDRDQLASVEAGAVLGDICAYANAGFTAERARQLSRLTGTHVPAGTGTEAASLRDSLCLLQKSYRFGSDSGIGQLAAAINRGDKTAVKTVFQQDFTDIEKRLLQSGEDYIAMLEEALAGYGRYLDLLQARAEPDLIIQAFNEYQLLCALREGPFGVAGLNERIEQFMQQKRKIHRHPHSRWYEGRPVMIARNDSALGLFNGDIGIALDRGQGTRVWFAMPDGNIKSVQPSRLPEHETTWAMTVHKSQGSEFDHAALILPSQRTPVVTRELVYTAVTRARRRLSLYADERILSAAIATRTERRSGLAALFSSRE |
| RNase II | MFQDNPLLAQLKQQLHSQTPRAEGVVKATEKGFGFLEVDAQKSYFIPPPQMKKVMHGDRIIAVIHSEKERESAEPEELVEPFLTRFVGKVQGKNDRLAIVPDHPLLKDAIPCRAARGLNHEFKEGDWAVAEMRRHPLKGDRSFYAELTQYITFGDDHFVPWWVTLARHNLEKEAPDGVATEMLDEGLVREDLTALDFVTIDSASTEDMDDALFAKALPDDKLQLIVAIADPTAWIAEGSKLDKAAKIRAFTNYLPGFNIPMLPRELSDDLCSLRANEVRPVLACRMTLSADGTIEDNIEFFAATIESKAKLVYDQVSDWLENTGDWQPESEAIAEQVRLLAQICQRRGEWRHNHALVFKDRPDYRFILGEKGEVLDIVAEPRRIANRIVEEAMIAANICAARVLRDKLGFGIYNVHMGFDPANADALAALLKTHGLHVDAEEVLTLDGFCKLRRELDAQPTGFLDSRIRRFQSFAEISTEPGPHFGLGLEAYATWTSPIRKYGDMINHRLLKAVIKGETATRPQDEITVQMAERRRLNRMAERDVGDWLYARFLKDKAGTDTRFAAEIVDISRGGMRVRLVDNGAIAFIPAPFLHAVRDELVCSQENGTVQIKGETVYKVTDVIDVTIAEVRMETRSIIARPVA |
| RNase III | GAMKITTWNVNSLNVRLPQVQNLLADNPPDILVLQELKLDQDKFPAAALQMMGWHCVWSGQKTYNGVAIVSRSVPQDVHFGLPALPDDPQRRVIAATVSGVRVINVYCVNGEALDSPKFKYKEQWFAALTEFVRDEMTRHGKLVLLGDFNIAPADADCYDPEKWHEKIHCSSVERQWFQNLLDLGLTDSLRQVHPEGAFYTWFDYRGAMFQRKLGLRIDHILVSPAMAAALKDVRVDLETRALERPSDHAPVTAEFDW |

# Table S2. Seventeen different REP sequences from natural *E. coli* gene sequence

| Number | Sequence (3'-5') | Secondary structure |
| --- | --- | --- |
| 1 | GCCGGAUGCCGGCGCCCAUAGCGCCUUAUCCGGCCUAC | ((((((((.(((((((...))))))))))))))).... |
| 2 | GCCGGAUGGCGGCGCGUAAUGCGCCUUAUCCGGCCUAC | (((((((((.((((((...))))))))))))))).... |
| 3 | GCCGGAUGCCGGCGCUACGUGCGCCUUAUCCGGCCUAC | ((((((((.(((((((...))))))))))))))).... |
| 4 | GCCGGAUGUCGGCGCCUGGCGCGCCUUAUCCGGCCUAC | ((((((((.(((((((...))))))))))))))).... |
| 5 | AUAGCUGAAUUGUUGGCUAU | .........(((((((((.. |
| 6 | AUGGGGCCCCGGUAAUCUUUCUAGUCGCCAAACUUGAAGAAGAUUAUCGGGGUUUUCGCUU | .(((((((..(((......))))))).)))((((((((((...))))))))))(((((((( |
| 7 | GGUGUUUGAGCCUUUCUGGUUCAGGCAAGACGCAGGUACCAGAAAUGCGAAGACCC | ((.((((.(((((((((((((((.(((...)))))).)))))))))))).)))))) |
| 8 | AGGUCUGCAUGAAGUACCUUAACACUACUGAUUGUAGCCU | (((.(((((..((((((.......))))))..)))))))) |
| 9 | GGCGCUUGAGGCUUUCUGCCUCAUGACGUGAAGGUGGUUUGUUGCCGUGUUGUGUGGCAGAAAGAAGAUAGCCC | ((.(((((..((((((((((((((((((...((((((....))))))))))))))))))))))))((.(((((( |
| 10 | AGGCAUCCCUAUGUCUAGUCCACAUCAGGAUAGCCU | ((((((....))))))((((((....))))))(((( |
| 11 | CGUGGUUAAUGCCACG | ((((((....)))))) |
| 12 | CGAUGCAGGGAGUUCUCUCCUCCCUGCAUCG | (((((((((((((.....))))))))))))) |
| 13 | UGUCCAUAGAAUCCUUUGUGAGGAGGUUCCUAUG | (((((((((((.(((((....)))))))).)))) |
| 14 | GGCGGAGUUCGACCGCC | ((((((.....)))))) |
| 15 | CGGGGUCGGACCGCUAUGCAGCGAACUGCAUAUUGCCCCG | ((((((((...))((((((((....)))))))).)))))) |
| 16 | CAUAGGAACCUCCUCACAAAGGAUUCUAUG | ((((((((((((......)))))))))))) |
| 17 | GUCGAUGCAGGGAGGAGAGAACUCCCUGCAUCGGC | (((((((((((((((.....))))))))))))))) |

# Table S3. Primers with different protective sequences for PCR

| Primer name | Sequence (5'-3') |
| --- | --- |
| Normal linear DNA-F | ccgagatctcgatcccgcgaaattaata |
| Normal linear DNA-R | atccggatatagttcctcctttcagca |
| GCGC-F-F | gcgcgcgcgcgcatatatattgcagcagcagtcgcttcacgttc |
| GGCC-F-F | ggggggccccccatatatattgcagcagcagtcgcttcacgttc |
| GCGC-F-R | gcgcgcgcgcgcatatatattccggatatagttcctcctttcag |
| GGCC-F-R | ggggggccccccatatatattccggatatagttcctcctttcag |
| GCGC-M-F | atatgcgcgcgcgcgcatattgcagcagcagtcgcttcacgttc |
| GGCC-M-F | atatggggggccccccatattgcagcagcagtcgcttcacgttc |
| GCGC-M-R | atatgcgcgcgcgcgcatattccggatatagttcctcctttcag |
| GGCC-M-R | atatggggggccccccatattccggatatagttcctcctttcag |
| GCGC-E-F | atatatatgcgcgcgcgcgctgcagcagcagtcgcttcacgttc |
| GGCC-E-F | atatatatggggggcccccctgcagcagcagtcgcttcacgttc |
| GCGC-E-R | atatatatgcgcgcgcgcgctccggatatagttcctcctttcag |
| GGCC-E-R | atatatatggggggcccccctccggatatagttcctcctttcag |


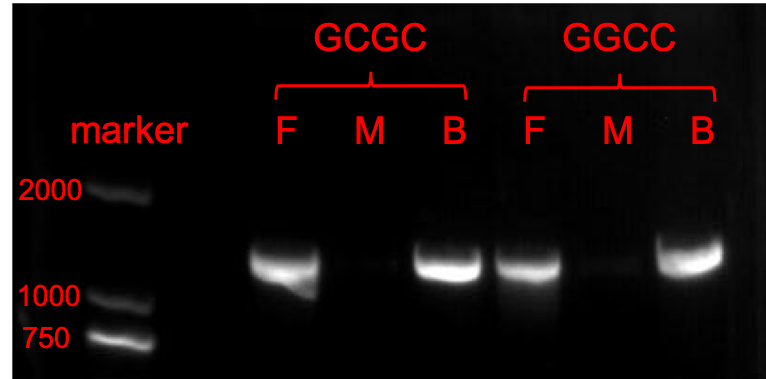


Figure S1. The agarose gel electrophoresis result of different linear templates with different protective sequences**.** The result showed that when the GC distribution was front and end, linear DNA was constructed well. However, when the GC distribution was middle, there were fewer PCR products.


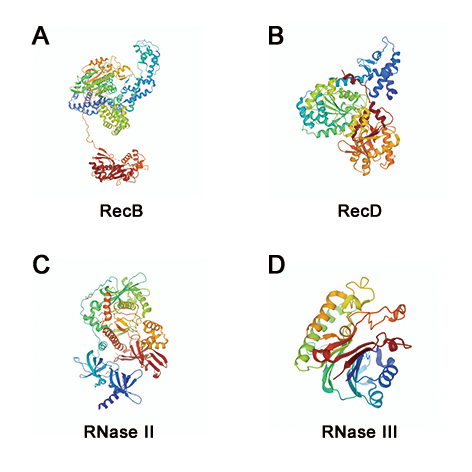


Figure S2. 3D structure of degrading enzymes used in this study**.** (A) 3D structure of RecB subunit. (B) 3D structure of RecD subunit. (C) 3D structure of RNase II. (D) 3D structure of RNase III.


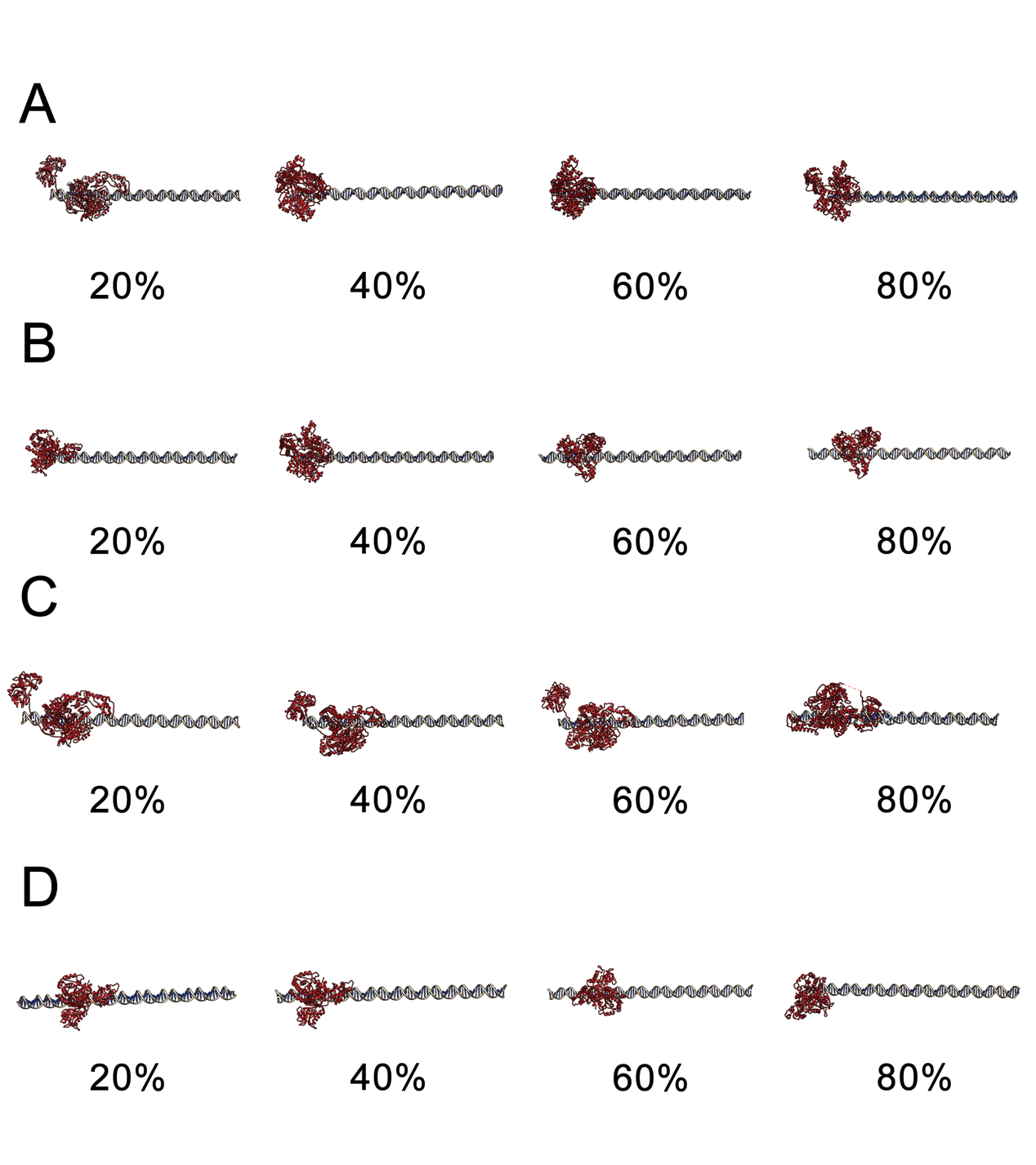


Figure S3. Simulation results of RecB subunit and RecD subunit with different protective sequences**.** (A) 3D structure of simulation results of RecB subunit with different protective sequences. These protective sequences were GCGC arrangement mode and had different GC content, as shown in the figure. (B) 3D structure of simulation results of RecD subunit with different protective sequences. These protective sequences were GCGC arrangement mode and had different GC content, as shown in the figure. (C) 3D structure of simulation results of RecB subunit with different protective sequences. These protective sequences were GGCC arrangement mode and had different GC content, as shown in the figure. (D) 3D structure of simulation results of RecD subunit with different protective sequences. These protective sequences were GGCC arrangement mode and had different GC content, as shown in the figure.


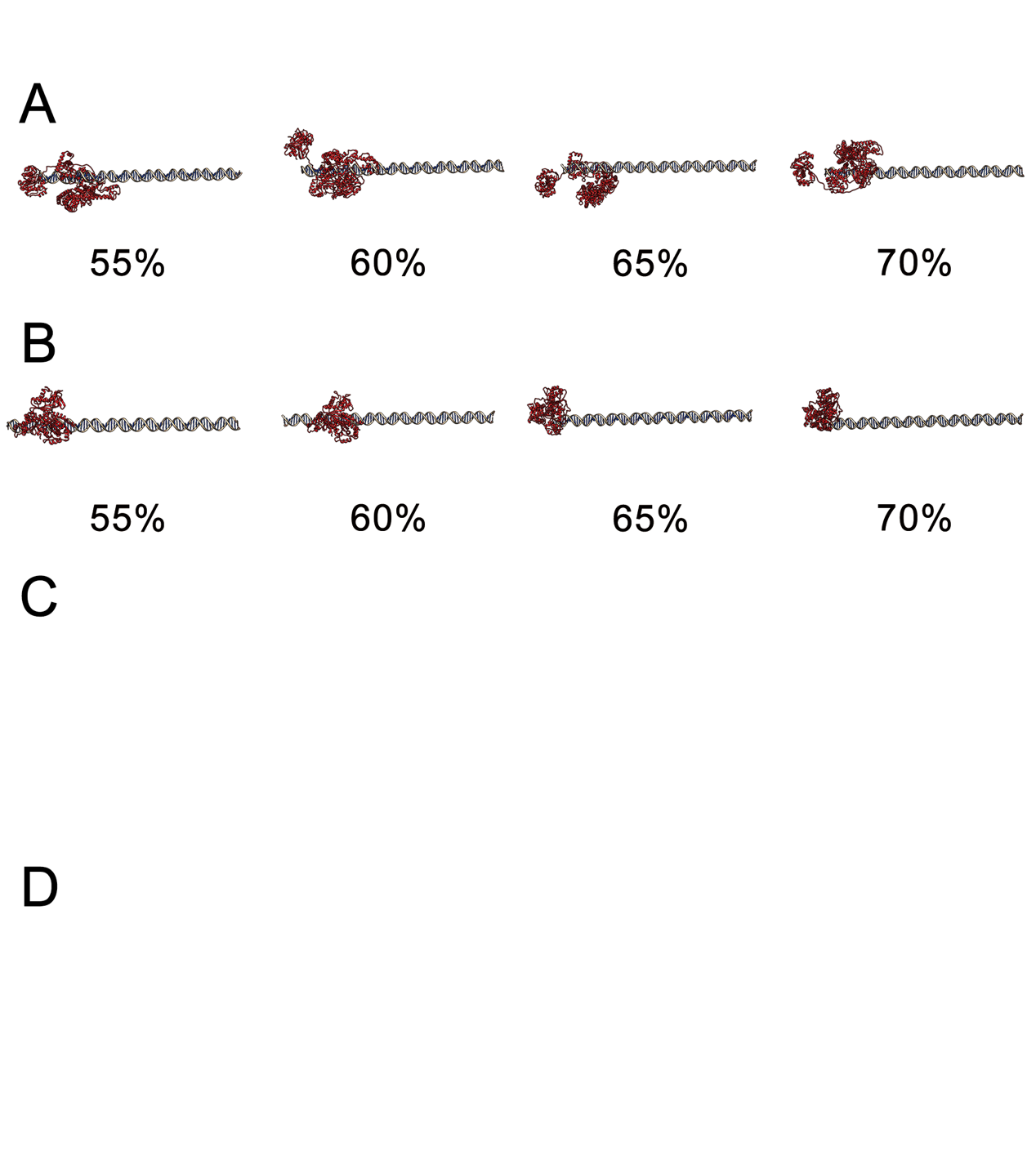


Figure S4. Simulation results of protective sequences with different GC content**.** (A) 3D structure of simulation results of RecB subunit with different protective sequences. These protective sequences were GGCC arrangement mode and had different GC content, as shown in the figure. (B) 3D structure of simulation results of RecD subunit with different protective sequences. These protective sequences were GGCC arrangement mode and had different GC content, as shown in the figure.


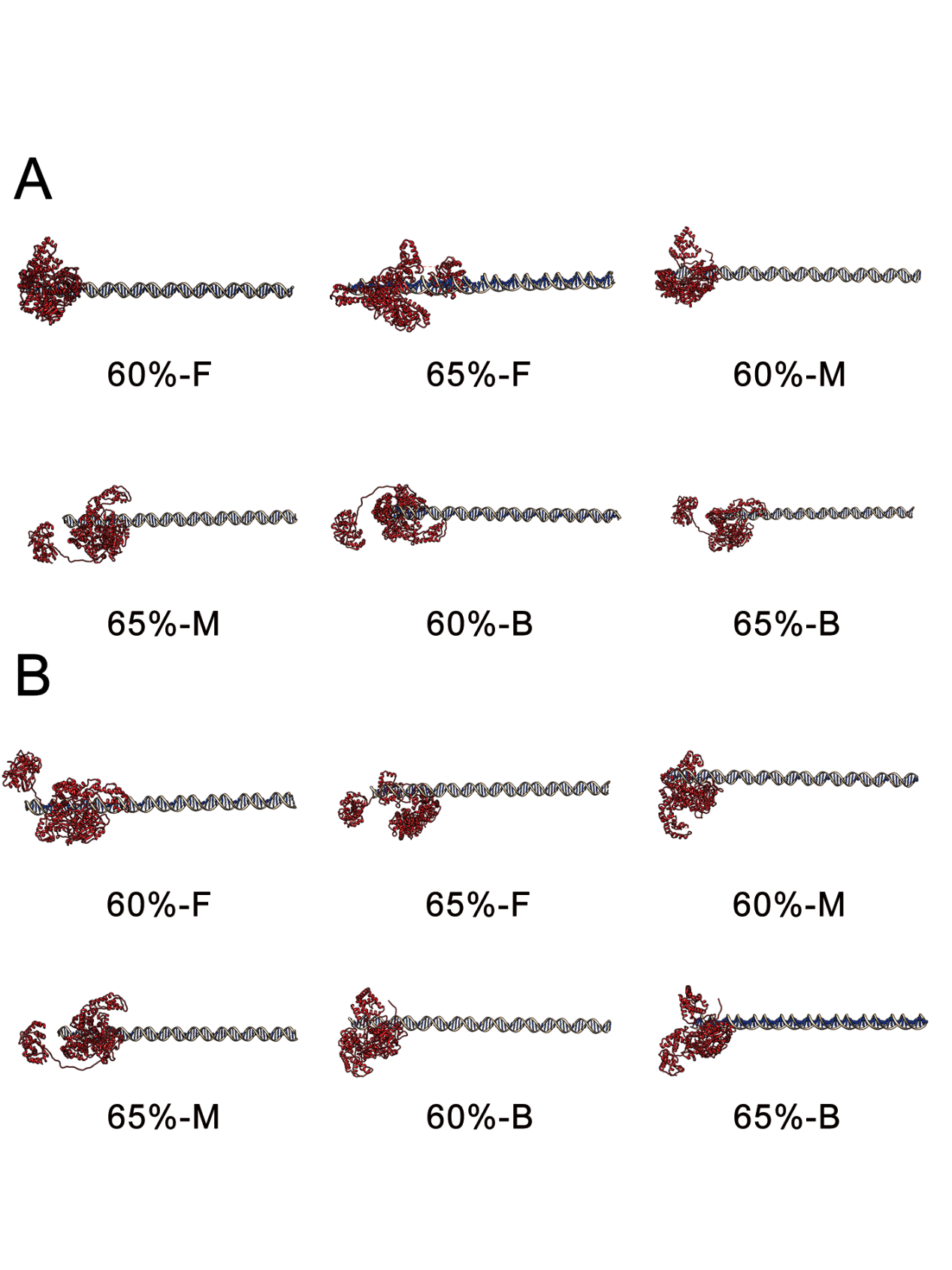


Figure S5. 3D structure of simulation results of RecB subunit with different protective sequences**.** The 60% and 65% meant different GC content. The letters *F*, *M* and *E* meant front, middle and back. (A) 3D structure of simulation results of RecB subunit with different protective sequences. These protective sequences were GCGC arrangement mode and had different GC content, as shown in the figure. (B) 3D structure of simulation results of RecB subunit with different protective sequences. These protective sequences were GGCC arrangement mode and had different GC content, as shown in the figure.


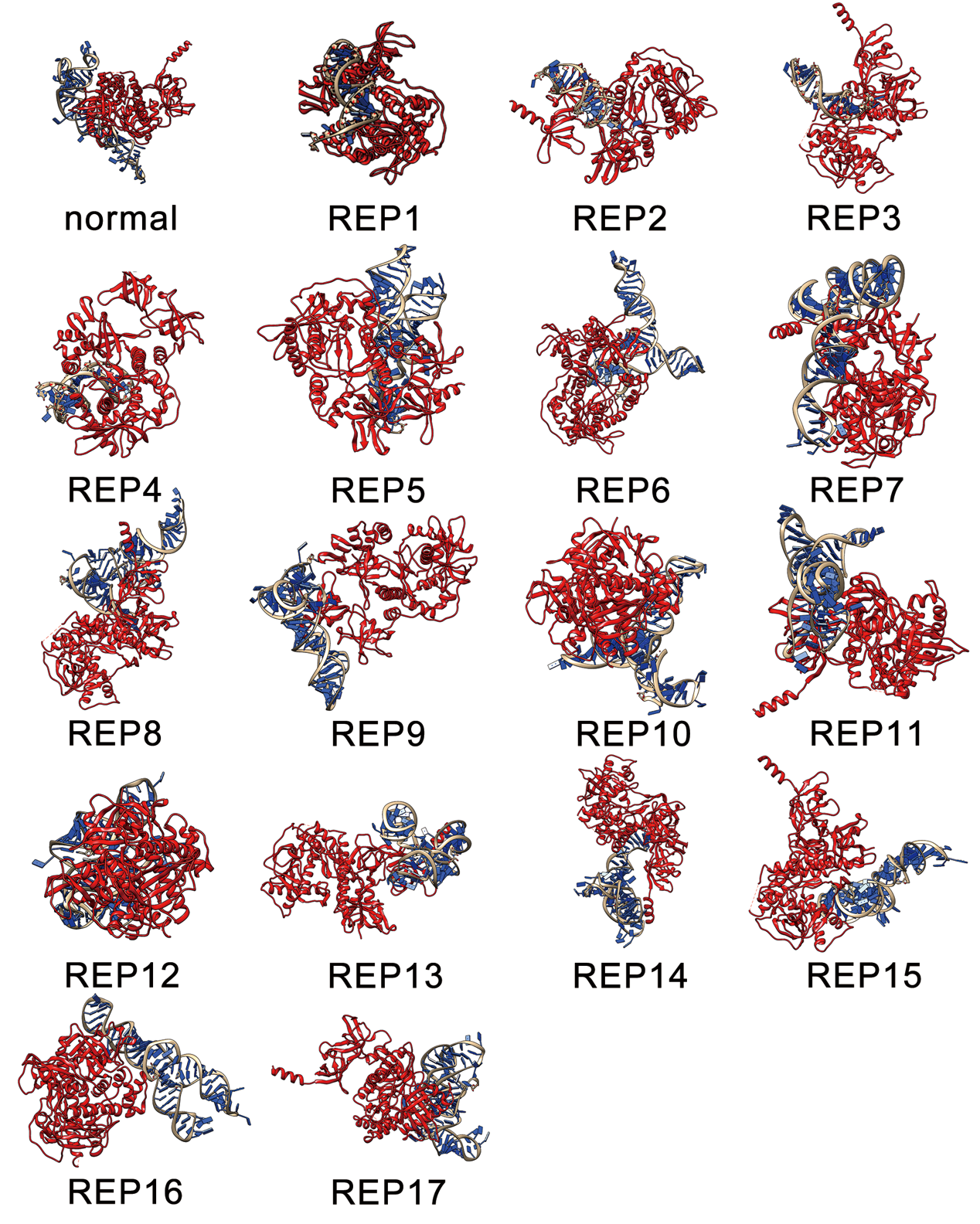


Figure S6. 3D structure of simulation results of RNase II and REP sequences**.** The numbers represented different REP sequences. The number *0* represented the control group without REP sequence.


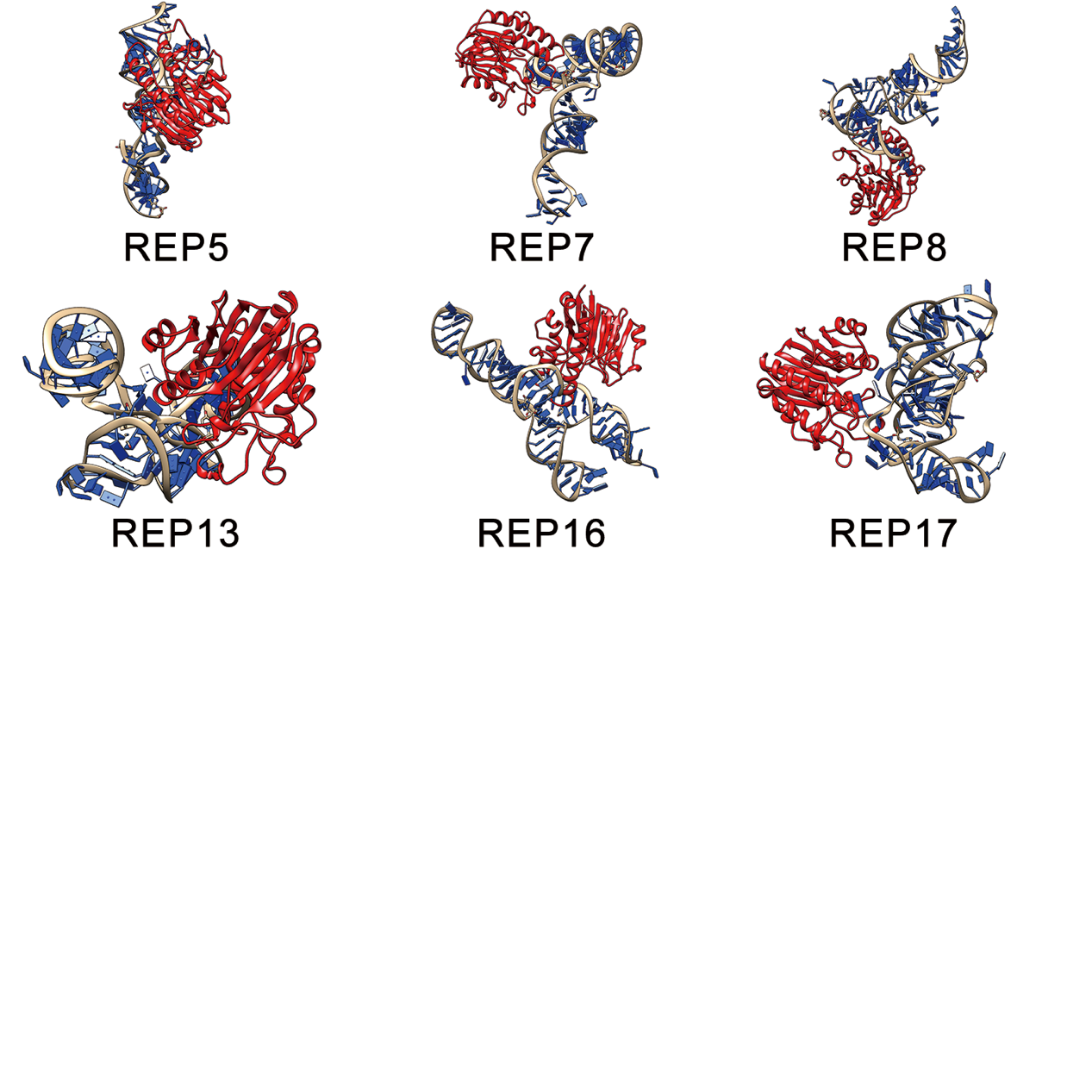


Figure S7. 3D structure of simulation results of RNase III and REP sequences**.** The numbers represented different REP sequences.

**
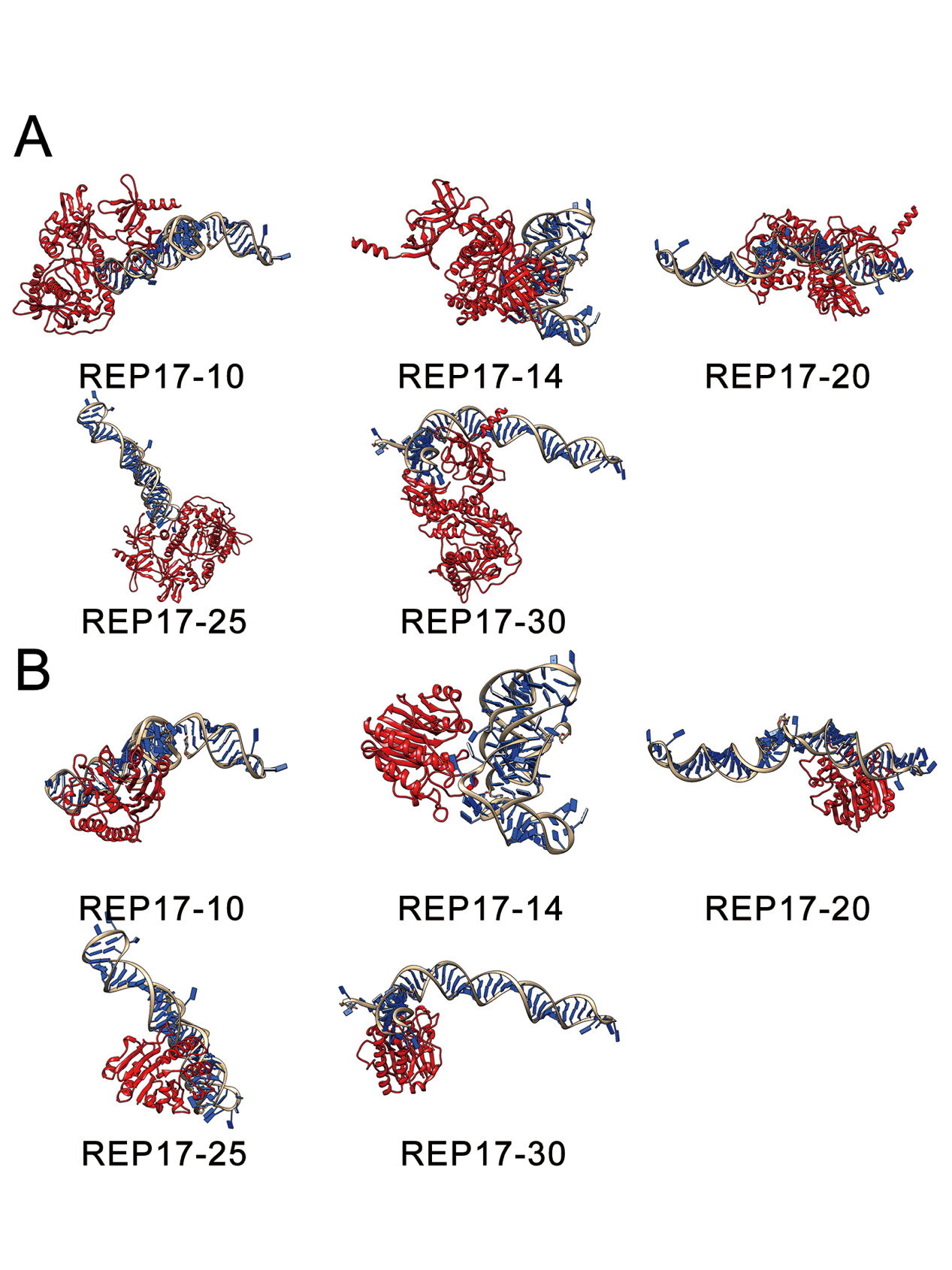
**

Figure S8. 3D structure of simulation results of REP sequences with different stem-loop structure length**.** The model REP sequence was the sequence number 17. (A) 3D structure of simulation results of RNase II with different REP sequences. These REP sequences had different lengthS, which ranged from 10 bp to 30 bp, as shown in the figure. (B) 3D structure of simulation results of RNase III with different REP sequences. These REP sequences had different lengths, which ranged from 10 bp to 30 bp, as shown in the figure.


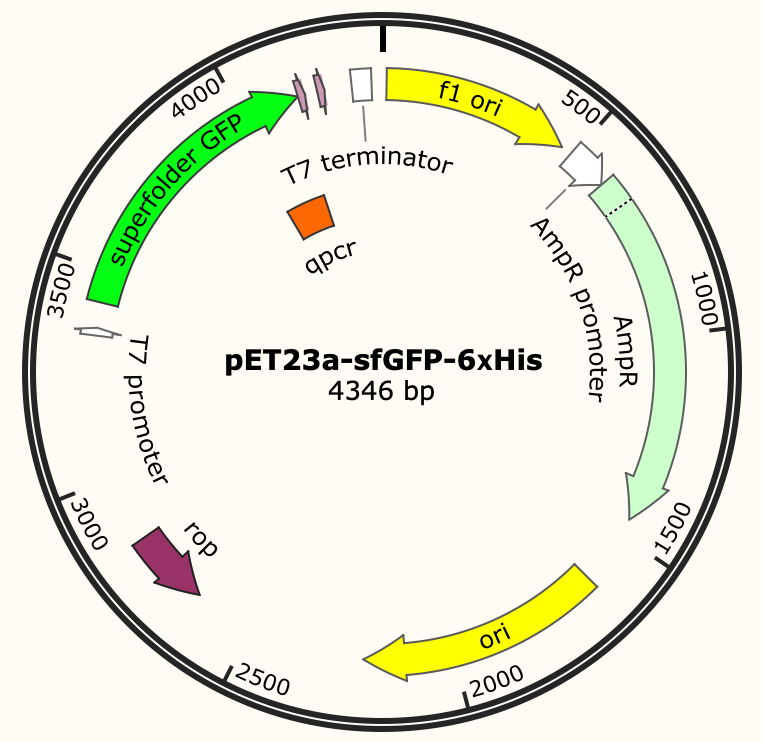


T7 promoter-sfGFP-6\242\252His-T7 terminator

TAATACGACTCACTATAGGGAGACCACAACGGTTTCCCTCTAGAAATAATTTTGTTTAACTTTAAGAAGGAGATATACATATGCGTAAAGGCGAAGAGCTGTTCACTGGTGTCGTCCCTATTCTGGTGGAACTGGATGGTGATGTCAACGGTCATAAGTTTTCCGTGCGTGGCGAGGGTGAAGGTGACGCAACTAATGGTAAACTGACGCTGAAGTTCATCTGTACTACTGGTAAACTGCCGGTACCTTGGCCGACTCTGGTAACGACGCTGACTTATGGTGTTCAGTGCTTTGCTCGTTATCCGGACCATATGAAGCAGCATGACTTCTTCAAGTCCGCCATGCCGGAAGGCTATGTGCAGGAACGCACGATTTCCTTTAAGGATGACGGCACGTACAAAACGCGTGCGGAAGTGAAATTTGAAGGCGATACCCTGGTAAACCGCATTGAGCTGAAAGGCATTGACTTTAAAGAAGACGGCAATATCCTGGGCCATAAGCTGGAATACAATTTTAACAGCCACAATGTTTACATCACCGCCGATAAACAAAAAAATGGCATTAAAGCGAATTTTAAAATTCGCCACAACGTGGAGGATGGCAGCGTGCAGCTGGCTGATCACTACCAGCAAAACACTCCAATCGGTGATGGTCCTGTTCTGCTGCCAGACAATCACTATCTGAGCACGCAAAGCGTTCTGTCTAAAGATCCGAACGAGAAACGCGATCATATGGTTCTGCTGGAGTTCGTAACCGCAGCGGGCATCACGCATGGTATGGATGAACTGTACAAACATCACCATCACCATCATTAAGTCGACAAGCTTGCGGCCGCACTCGAGCACCACCACCACCACCACTGAGATCCGGCTGCTAACAAAGCCCGAAAGGAAGCTGAGTTGGCTGCTGCCACCGCTGAGCAATAACTAGCATAACCCCTTGGGGCCTCTAAACGGGTCTTGAGGGGTTTTTTG

Figure S9. The pET23a-sfGFP-6\242\252His plasmid map and the sequence information**.** The sfGFP was regulated under the bacteriophage derived T7 promoter and terminator in the pET-23a backbone.


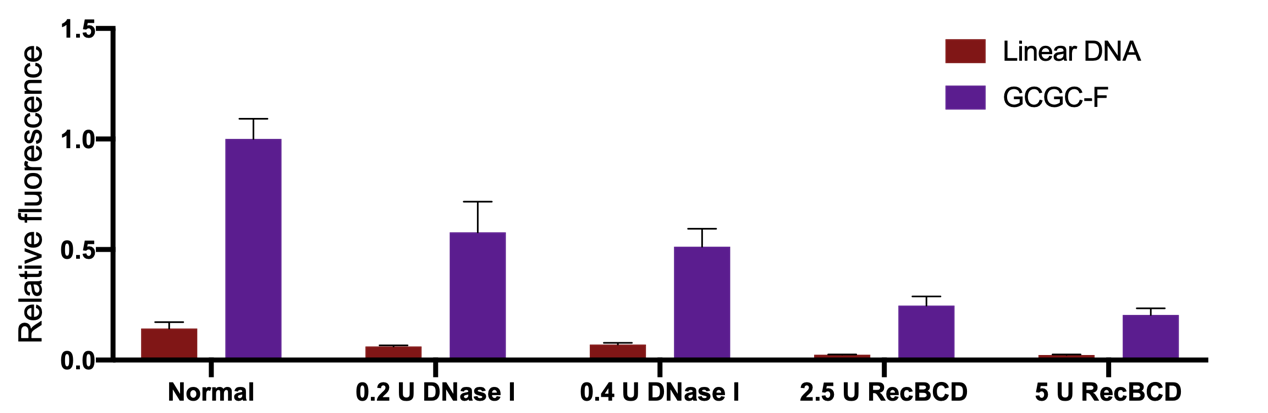


Figure S10. The protein expression level of the LET-based cell-free system with additional nucleases**.** The normal meant no additional nuclease had been added to the cell-free gene expression system. Linear DNA meant the linear expression templates without protective sequences. The GCGC meant protective sequences with GCGC arrangement mode were added to the linear expression templates. The letter F meant GC distribution mode was front. The enzyme activities of DNase I used were 0.2 U and 0.4 U. The enzyme activities of the RecBCD complex used were 2.5 U and 5 U. These nucleases were purchased from New England Biolabs (Beijing, China).


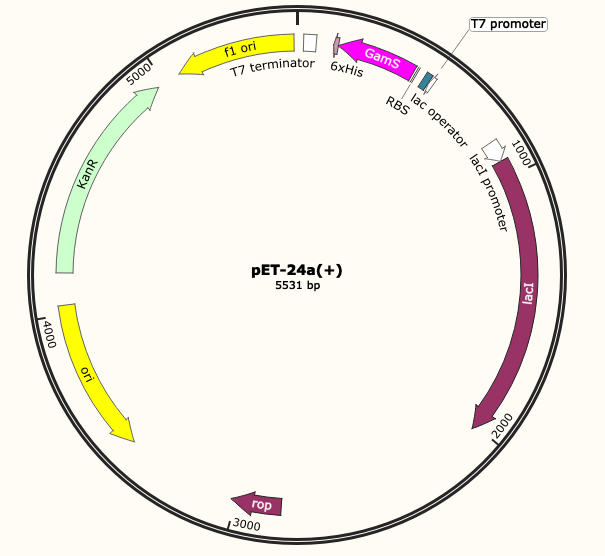


T7 promoter-GamS-6xHis-T7 terminator

TAATACGACTCACTATAGGGGAATTGTGAGCGGATAACAATTCCCCTCTAGAAATAATTTTGTTTAACTTTAAGAAGGAGATATACATATGGGAAACGCTTATTACATTCAGGATCGTCTTGAGGCTCAGAGCTGGGCGCGTCACTACCAGCAGCTCGCCCGTGAAGAGAAAGAGGCAGAACTGGCAGACGACATGGAAAAAGGCCTGCCCCAGCACCTGTTTGAATCGCTATGCATCGATCATTTGCAACGCCACGGGGCCAGCAAAAAATCCATTACCCGTGCGTTTGATGACGATGTTGAGTTTCAGGAGCGCATGGCAGAACACATCCGGTACATGGTTGAAACCATTGCTCACCACCAGGTTGATATTGATTCAGAGGTACTCGAGCACCACCACCACCACCACTGAGATCCGGCTGCTAACAAAGCCCGAAAGGAAGCTGAGTTGGCTGCTGCCACCGCTGAGCAATAACTAGCATAACCCCTTGGGGCCTCTAAACGGGTCTTGAGGGGTTTTTTG

Figure S11. The pET23a(+)-GamS-6xHis plasmid map and the sequence information**.** The GamS was regulated under the bacteriophage derived T7 promoter and terminator in the pET-23a(+) backbone.


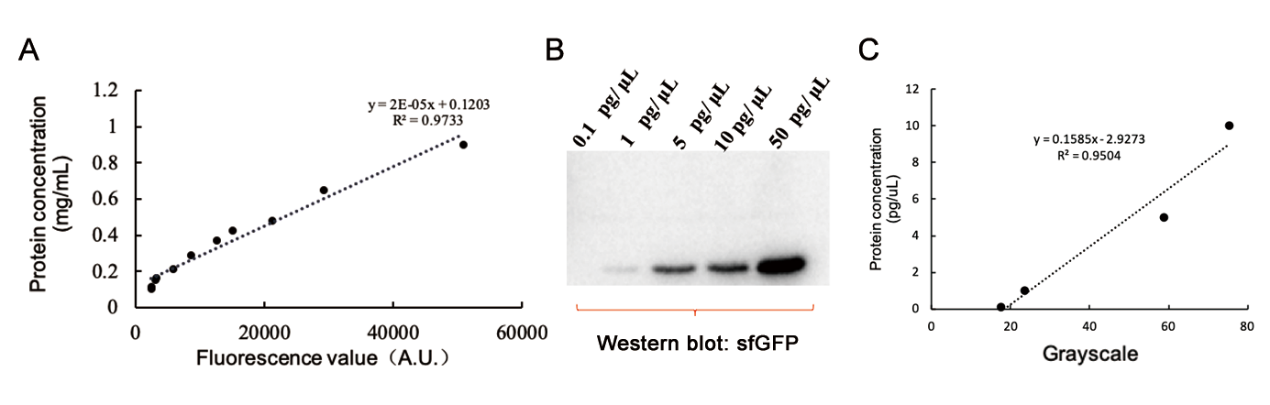


Figure S12. The fluorescent value could represent the protein expression level**.** (A) Standard curve of sfGFP. The sample was diluted 20 times. The fluorescent value of sfGFP increased linearly with the concentration of protein, which indicated that the protein expression level could be characterized by the fluorescence, and the influence of the environment could be ignored. (B) The results of western-blot. The grayscale value increased linearly with the concentration of protein. (C) The protein concentration and grayscale value plot. The grayscale value increased linearly with the concentration of protein.


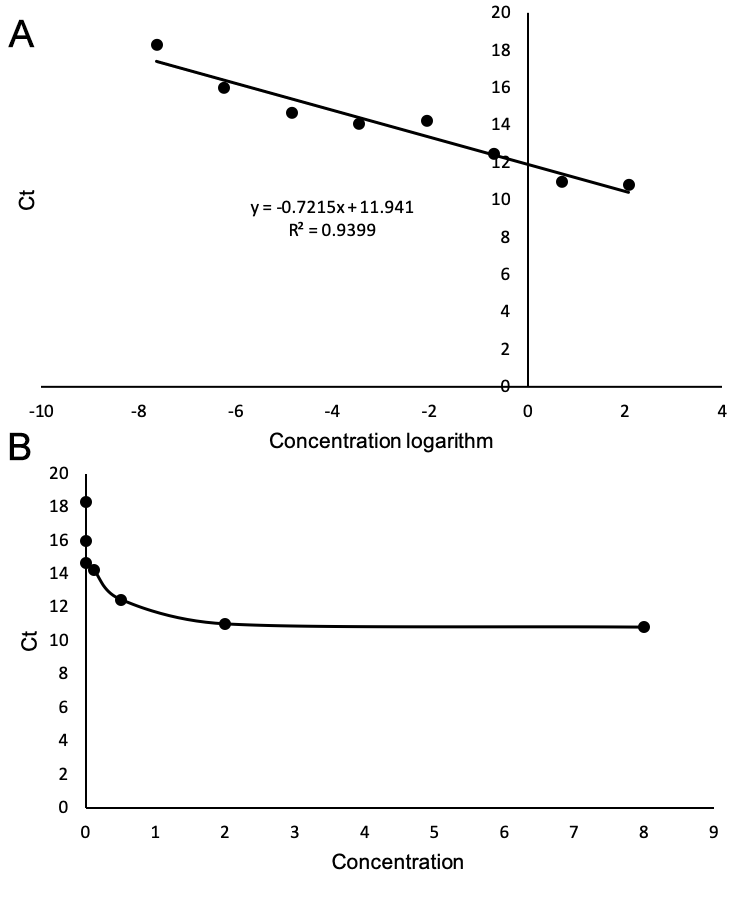


Figure S13. Standard curve of sfGFP cDNA concentrations and CT values**.** The PCR product of sfGFP was amplified by the same primers as qPCR for the standard curve to relate the sfGFP cDNA concentrations with CT values read by ABI 7300 Real-Time PCR system. (A) The CT and concentration logarithm plot. (B) The CT and concentration plot.
